# A 95-Year-Old Concrete Arch Bridge: From Materials Characterization to Structural Analysis

**DOI:** 10.3390/ma14071744

**Published:** 2021-04-01

**Authors:** Andrzej Ambroziak, Maciej Malinowski

**Affiliations:** Faculty of Civil and Environmental Engineering, Gdansk University of Technology, 11/12 Gabriela Narutowicza Street, 80-233 Gdańsk, Poland; maciej.malinowski@pg.edu.pl

**Keywords:** structural analysis, bridge engineering, reinforced concrete, mechanical properties

## Abstract

The structural analysis of a 95-year-old concrete arch bridge located in Jagodnik (Poland) is performed in this paper, in order to check its behavior under today’s traffic loads. The mechanical properties of both the concrete and the reinforcement are investigated by testing cores and bar stubs extracted from the bridge. Structural analysis confirms that the bridge meets today’s load requirements in terms of bearing capacity, serviceability state, and that the adopted structural improvements (a new deck slab on top of the existing structure and a layer of mortar to protect the surface of the old concrete) are effective. In this way, the 95-year-old arch bridge was given a new life. The structural improvements show how combining numerical modelling and laboratory tests can contribute to the preservation of an old—though fairly simple—and valuable structure, otherwise destined to demolition, with both environmental and economic benefits.

## 1. Introduction

Arch bridges are one of the most popular types of bridges. At present, there are over 40 concrete arch bridges in the world with a span of greater than 200 m [1]. Concrete application in the development of arch bridges has a long and interesting history [2]. In most countries, there are old bridges that require maintenance, renovation, or reconstruction [3]. In the literature, it is possible to find many interesting investigations related to the process of testing and repairing old concrete bridges [4,5,6,7,8,9,10,11,12,13,14,15] or to the structural analysis of old bridges [16,17,18,19,20,21,22]. Reconstruction and renovation of old bridge structures and adaptation to new traffic loads are complex issues often requiring not only the experience of civil engineers, but also that of the scientific community. Before dealing with the technical conditions and bearing capacity of any given bridge structure, a detailed inspection is a must. The code provisions concerning the original materials like mechanical, chemical, and also physical properties are required for proper assessment of conditions of old structures to reflect their real technical conditions. The scope of material tests should be adapted to the specificity of the construction and location of the bridge structure. The design team often faces the problem of limited or even lacking original documentation. It is then necessary to make a detailed inspection which is instrumental in defining the scope of the reconstruction or repair process, as well as in formulating a numerical model. New technics, like laser scanning, photogrammetry techniques and ground penetrating radar [23,24,25,26,27,28,29], have been increasingly used in terms of monitoring, inventory control and structural inspection. New tools and techniques are very helpful in the technical and theoretical assessment of old bridge structures.

The present study is aimed at the structural analysis of the 95-year-old concrete arch bridge based on mechanical properties measured by means of laboratory tests. Structural analysis was a part of an expert opinion, required to check whether the old arch bridge has an adequate bearing capacity face to today’s traffic loads, in order to extend its service life. The present paper supplements and extends the investigations performed by Ambroziak et al. [30] on the design stage. In the present paper, new concrete samples taken from the old concrete arch bridge during its reconstruction were tested in a laboratory and the results of the tests on the original steel reinforcement are presented. The results about the internal forces under design loads are determined and the maximum stresses in both the concrete and the reinforcement are evaluated and compared with the design stresses, and the displacements are checked with reference to those specified for the service limit state. The paper provides scientists, engineers, and designers the example of structural analysis results and experimental assessment of the 95-year-old concrete arch bridge.

## 2. Materials and Methods

**Figure 1:**
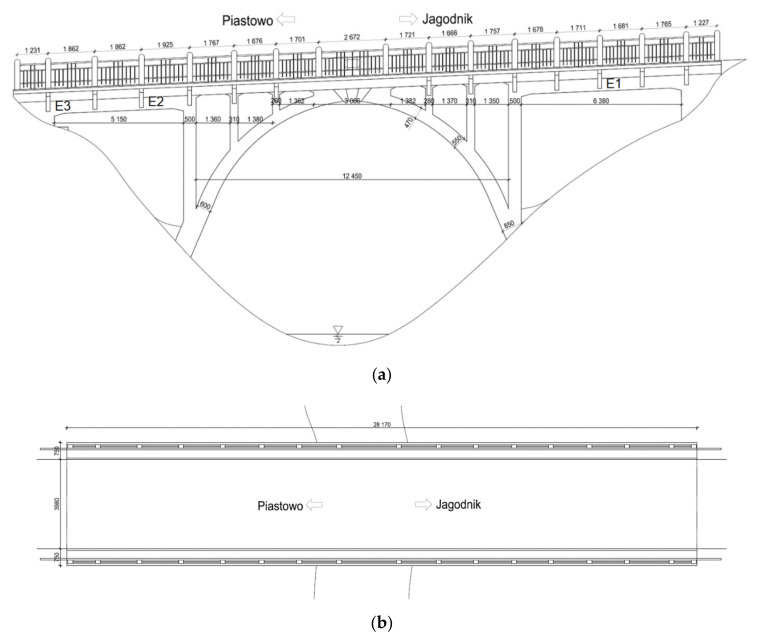
Jagodnik arch bridge before reconstruction (dimension units—mm): (**a**) Side view; (**b**) view from above.

The old bridge investigated (see Figure 1) is an arch bridge built in 1925 close to the city of Elbląg (village Jagodnik in Poland) above the Kumiel river. Karl Metzger & Co. building company [31] was responsible for the construction of the bridge, which consists of a reinforced-concrete slab monolithically connected to a reinforced-concrete arch with a span equal to 12.95 m.

**Figure 2:**
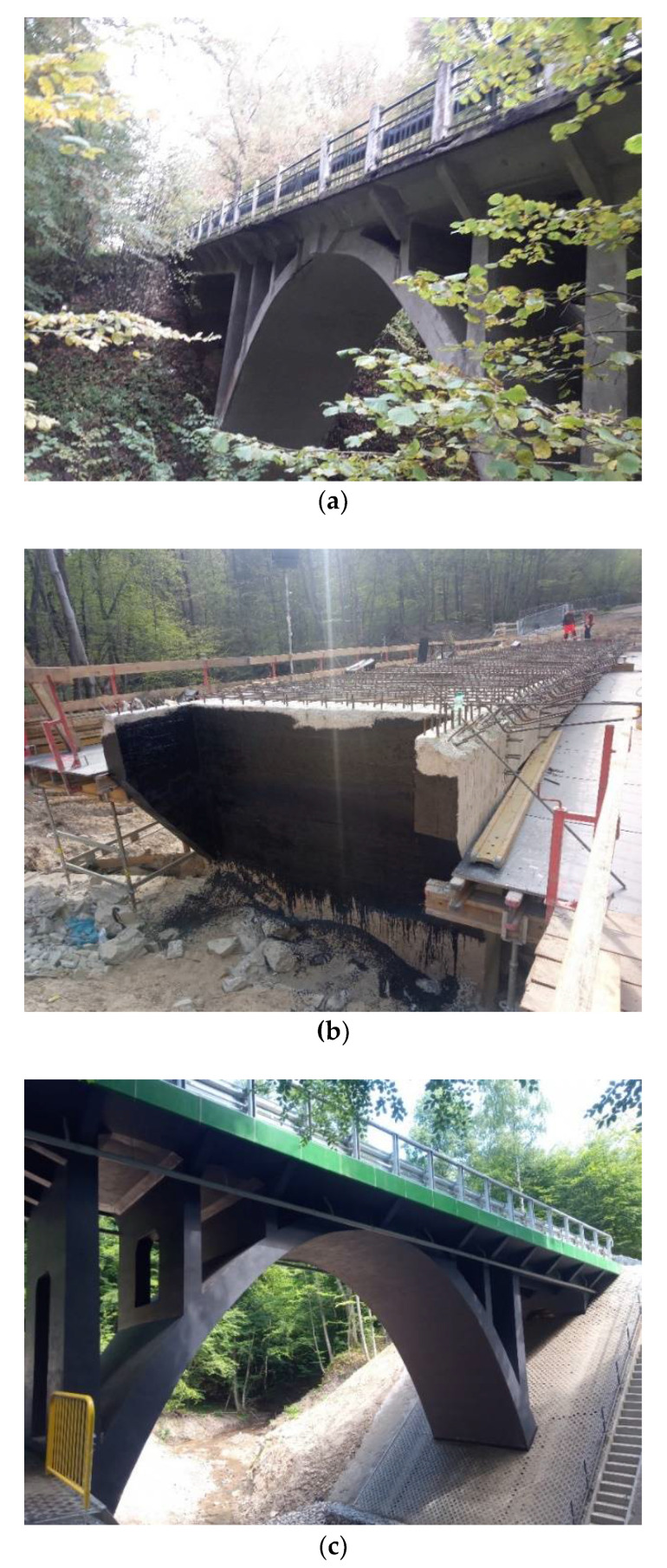
Jagodnik arch bridge: (**a**) Before; (**b**) during; and (**c**) after reconstruction.

**Figure 3:**
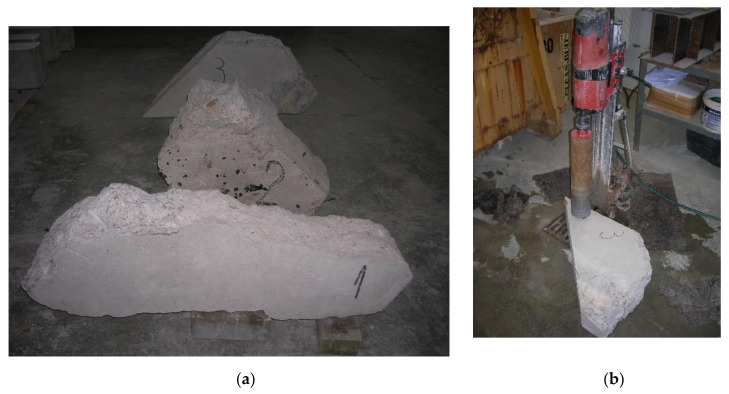
Preparation of the concrete cores: (**a**) Fragments of old concrete; (**b**) drilling process.

Before the reconstruction process of the 95-year-old concrete arch bridge (see Figure 2) a few fragments of the old concrete were delivered to a laboratory (see Figure 3a) and seven concrete cores were extracted by means of a borehole diamond drill machine (see Figure 3b). After finishing, the length-to-diameter ratio L/D was 1 (L = D = 100 mm; see EN 12504-1 [32] standard), as the thickness of the old structural concrete members was close to 15–16 cm. The cores were marked with the indication of their location and specimen number (location number_specimens number, e.g., 1_2, 2_1). Stubs of steel bars were tested as well. The concrete fragments E1, E2, and E3 (Figure 3a) were taken from locations indicated in Figure 1a.

The concrete cylinders were tested in compression to the failure according to EN 12390-3 standard [33]. Concrete dry density was determined according to method guidelines in EN 12390-7 standard [34], after drying the specimens in a ventilated oven (T = 105 ± 5 °C) until mass stabilization (not more than 0.2% mass variation with respect to the original mass). The density was derived after cooling down to room temperature in dry conditions. The tests in uniaxial tension of steel reinforcements were performed in accordance with ISO 6892-1 standard [35].

## 3. Laboratory Test Results and Discussion

### 3.1. Reinforced Steel Tensile Tests

**Figure 4:**
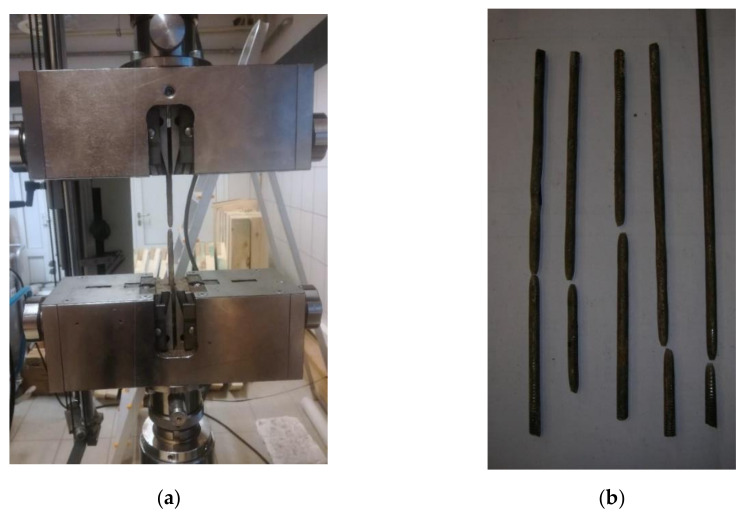
Uniaxial tensile tests: (**a**) Test setup; (**b**) steel bars after failure.

**Figure 5:**
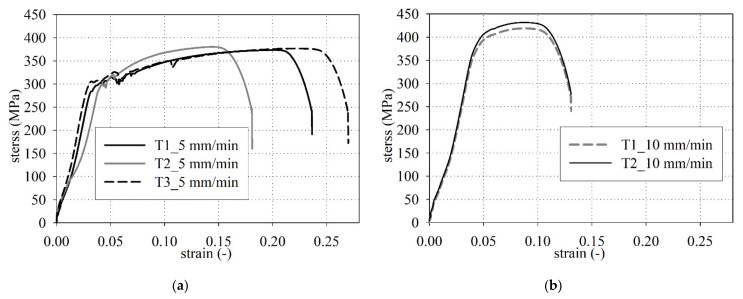
Stress–strain curves in tension: Test repeatability for two different displacement rates, 5 mm/s (**a**) and 10 mm/s (**b**).

The reinforcement smooth steel bars of a 6 ± 0.1 mm diameter were taken from the old arch bridge and subjected to uniaxial tension by means of the computer-controlled Zwick Z400 testing machine (ZwickRoell GmbH & Co. KG, Ulm, Germany), see Figure 4. The length of the sample between the grips is 100 mm and the displacement rate is 5 mm/min. All tests were performed at room temperature (about 20 °C) and were carried out up to specimen failure. Three specimens were chosen for uniaxial tensile tests. During tensile tests, the results were recorded sampling every 10 μm (traverse displacement interval), 20 ms (time interval) and 1 N (force interval). The tests in tension on the bar stubs were carried out in accordance with ISO 6892-1 standard [35]. The engineering strain at rupture show range from 15% to near 25%, while the ultimate tensile strength covers the 374–380 MPa interval. The investigated steel rebars are characterized by clear yield strength, strain hardening, and necking range at the stress–strain curves, see Figure 5a. The yield strength was determined in laboratory tests equals 291 ± 7 MPa. The yield strength is defined as the lowest value of stress during plastic yielding, ignoring any initial transient effects. The stress is obtained by dividing the force by the original cross-sectional area of the steel bars.

The Regulations on the Construction and Maintenance of Road Bridges [36] approved by the Polish Minister of Public Works (the ordinance of 9.XI.1925 no. XIII-1386) state the yield strength of steel rebars not to be less than 294 MPa (3000 kg/cm^2^). The yield strength (291 ± 7 MPa) specified in laboratory tests corresponds to the guidelines issued in the arch bridge construction time. The Post-Second World War standard PN-B-195 [37] made it possible to apply three types of steel bars of variable yield strengths equals: 196 MPa (2000 kg/cm^2^), 235 MPa (2400 kg/cm^2^), and 353 MPa (3600 kg/cm^2^). The S500 steel grade of 500 MPa characteristic yield strength is an abundant concrete reinforcement on bridge and building sites today.

Additionally, two bar stubs were tested at a displacement rate equal to 10 mm/min (Figure 5b). The elastic-viscous behavior of the material is evident (the higher the strain rate, the higher the strength at yielding [38]). In Figure 5b, the initial S-shaped loading branch was probably due to grip sliding, while the subsequent mostly-linear branch (stress comprised between 150 and 350 MPa) is related to concrete linear-elastic behavior. Finally, nonlinearity starts at 350 MPa.

### 3.2. Uniaxial Compressive Tests and Dry Density

The uniaxial compressive experimental tests were conducted on the Advantest 9 C300 KN mechanical testing machine. The experiments were performed to the failure of the concrete cylinder specimens and were used at a constant rate of loading with a range of 0.6 MPa/s according to EN 12390-3 [33]. The uniaxial compression test results of compressive strength for cylindrical samples are presented in Table 1. These results are accompanied by the results of the previous investigation. The mean compressive strength of cylindrical samples is equal to 20.5 ± 1 MPa while the median is equal to 19.7 MPa. The strengths of normal-weight concrete determined on cored specimens with a diameter of 100 mm have no different from those for standardized cube specimens with a 150 mm side length [39] as opposed to lightweight aggregate concrete [40]. It is worth noting that concrete tends to behave as a homogeneous material as long as the sample size is a multiple of the maximum aggregate size, which implies for the diameter of concrete cores to be at least three times larger than the maximum aggregate size. The strength results determined for the concrete cores *f*_ck,is,cycl 100_ according to standard EN 12504-1 [32] are identical to the cube strength of 15 × 15 × 15 cm concrete specimens, thus *f*_ck,is,cube_ = *f*_ck,is, cycl 100_ = 20.5 ± 1 MPa. The mean compressive strength of old concrete cylindrical samples slightly exceeds the value presented by Ambroziak et al. [30] (18.8 ± 0.7 MPa) in early investigations.

The concrete had large variations in compressive strength ranging from 14.9 MPa to 29.7 MPa, see Table 1. To properly perform the structural analysis of old concrete structures, it is necessary to evaluate the old concrete compressive strength. The characteristic in-situ compressive cube strength *f*_ck,is,cube_ according to EN 13791 [41] standard can be determined as:

(1) $$f_{\mathrm{ck},\mathrm{is},\mathrm{cube}}=\min\left\{\begin{array}{c} f_{m(n),\mathrm{is}}-k_{n}\cdot s\\ f_{\mathrm{is},\mathrm{lowest}}+M \end{array}\right\}=\min\left\{\begin{array}{c} 20.5-1.81\cdot 1\\ 14.9+2 \end{array}\right\}=16.9\;\mathrm{MPa}$$

where *f*_m(n),is_ is the mean in-situ compressive strength of *n* = 16 test results, *f*_is,lowest_ is the lowest in-situ compressive strength test results, *k*_n_ is the factor depends on the number of tests results (*k*_n_ = 1.81 for tests results equal to 16, see EN 13791 [41], *s* = 1 MPa is the standard deviation of in situ compressive strength, *M* = 2 MPa is the value of margin depend on value of *f*_is,lowest_ (see EN 13791 [41], 12 MPa ≥ *f*_is,lowest_ < 16 MPa). Ambroziak et al. [30] in their earlier research for old concrete set the same value of the characteristic in-situ compressive cube strength equal to 16.9 MPa. The decisive condition for determining the characteristic in-situ compressive cube strength is governed by the value of the lowest in-situ compressive strength. The differences in single cylindrical compressive strength exhibit a non-homogenous distribution of concrete strength in the old concrete arch bridge.

**Figure 6:**
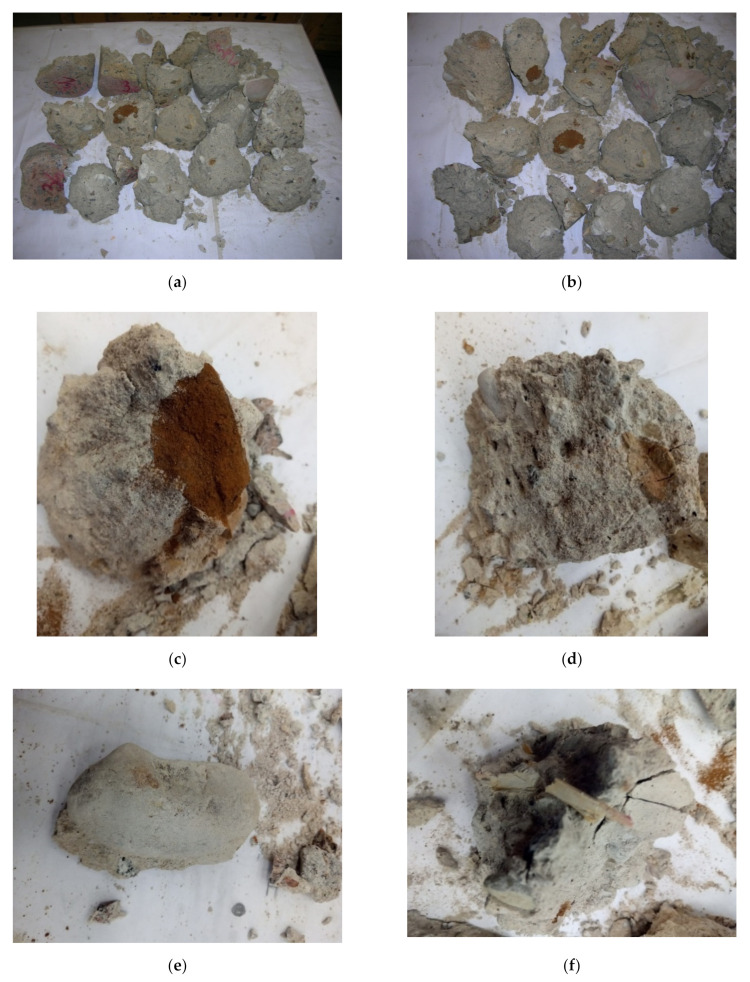
Concrete specimens after uniaxial compressive tests: (**a**,**b**) Form of specimens failure; (**c**) dirty fine aggregate; (**d**) clay inclusion; (**e**) large stone; (**f**) timber inclusions.

High compressive strength variation among individual concrete specimens is produced by impurities that were identified after uniaxial compressive tests, see Figure 6a–f. Parts of timber, piece of clay, coarse aggregates (large stones) with cavities and pores were detected in some concrete cores. The maximum aggregate size used in the old concrete mix is up to 20 mm. In a single individual case, the maximum aggregate size was up to 50 mm (see Figure 6e). Additionally, a wide scatter in compressive strength may be affected by the proportion of cement and aggregate (sand to gravel volumetric ratios) for the old concrete mix preparation. Concrete strength may also be affected by different climatic conditions in the course of placement [42]. The specimens were crushed or got separated along a slanted surface and columnar vertical cracking through both ends with no well-formed cones was observed. Generally, the failure mode of core specimens (see Figure 6a,b) was typical and fulfills requirements guidelines in EN 12390-3 [33] standard.

The reinforced concrete structural guideline [43] issued in January 1916 by the German Committee for Structural Concrete specified two main concrete strength classes 14.7 MPa (150 kg/cm^2^) and 17.7 MPa (180 kg/cm^2^) for the erection of concrete structures. This guideline has been applicable till September 1925, i.e., while German standard DIN 1045 [44] was introduced. On the other hand, while the 15–18 MPa compressive strength is required the Hennebique recommends a mixture consisting either of a single part cement, two parts sand and four parts gravel or of one part cement, three parts sand and five parts of gravel [45]. The Regulations on the Construction and Maintenance of Road Bridges [36] made it possible to forecast the cube compressive strength of concrete related to the amount of cement to 1 m^3^ aggregate in concrete mixes, see Table 2. According to these regulations, the amount of water in the concrete mix should be appropriate to locate the mixed concrete in the formwork, or to hand-knead the compacted concrete ball. In the hand-mixing case the amount of cement should be increased by 5%, while consistency of a liquid mix is regarded, the 10% increment is anticipated.

The Polish standard PN-B-195 [37] specified the forecast compressive strength of concrete with regard to the amount of cement in a 1 m^3^ concrete mix, the volume ratio of aggregates and the consistency of the ready concrete mix, see Table 3.

According to the PN-B-195 standard [37] the characteristic strength of concrete, equal 19.6 MPa (200 kg/cm^2^) may be achieved by use of 400 kg of cement with a 1:2 ratio of sand to gravel parts in 1 m^3^ of finished concrete of a rammed concrete consistency. The standard PN-B-195 [46] in its early version of 1934 specified the strength equal to 16.7 MPa (170 kg/cm^2^) with the same amount of cement. The lack of clear and detailed water dosage guidelines produced variable compressive strengths of old concrete structures. The standard PN-B-195 [37] emphasized that the amount of water should be limited in ready concrete mixes of 0 MPa (0 kg/cm^2^, see Table 3) concrete strength class when the liquid concrete mix consistency is assumed. The water-to-cement ratio is defined in the present standards and guidelines regarding concrete mixes, specifying the proper amount of water in concrete mix for a prescribed concrete strength class.

The method specified in EN 12390-7 [34] standard is applied for determining the dry density of 95-year-old concrete. The tested specimens were dried in a ventilated oven at 105 ± 5 °C until the mass relative decrement reaches 0.2%. Before weighing each specimen was cooled to near room temperature in a dry airtight vessel. The mean dry density value is equal to 2164 ± 9 kg/m^3^ while the median is equal to 2173.5 kg/m^3^, see Table 1. According to the EN 206 standard [47] and the ACI 318-19 code [48], the investigated old concrete satisfies the conditions for the normal-weight concrete category.

### 3.3. Modulus of Elasticity and Durability

The secant modulus of elasticity in the range 0 to 40% of the ultimate strength (according to EN 1992-1-1 [49] standard) is assumed 22,890 MPa (mean value of the modulus of elasticity, see Ambroziak et al. [30]) with regard to structural analysis of old arch concrete bridge. The ASTM C469 M standard [50] guideline was used to determine the modulus of elasticity. Diamond-drilled concrete cores with a length to diameter ratio of 1.50 were used in a compressometer device to measure the static modulus of elasticity. The regulations [36], applicable in time of the bridge erection indicated that the concrete compressive strength greater or equal to 13.73 MPa corresponds to the modulus of elasticity 14,715 MPa (150,000 kg/cm^2^). The modulus of elasticity assumed for investigated old concrete is 1.55 times greater than the value specified in regulations [36], possibly due to various aggregates types in concrete mixes.

Based on the early experimental investigation performed by Ambroziak et al. [30] it can be concluded that the 95-year-old concrete has good freezing resistance. The chloride content of the old concrete did not exceed 0.2% by mass of cement; thus, the old concrete arch bridge was not exposed to chloride attack. The pH values for the old concrete indicated that there is no corrosion of the steel rebar’s. Nevertheless, the old concrete has large variations in depth of the carbonated zone ranging from 20 to 55 mm. Despite the large depth of the carbonated zone, the pH of the old concrete is still in the safety range.

## 4. Structural Analysis

### 4.1. Description of FEM Model

**Figure 7:**
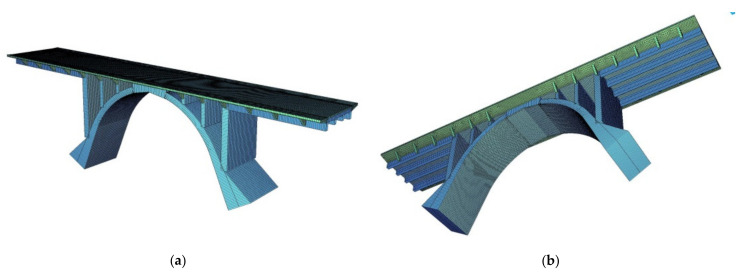
FEM model: (**a**,**b**) 3 D views.

**Figure 8:**
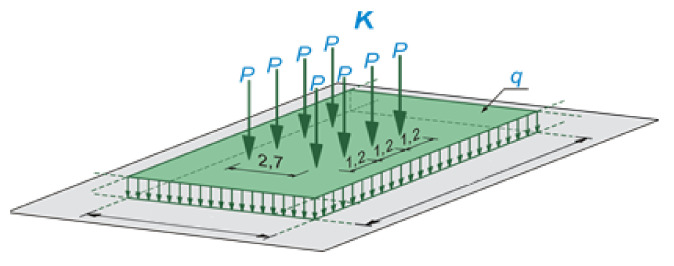
Traffic loads class C (q and K types) according to standard PN-S-10030 [56].

The three-dimensional finite element model of the 95-year-old concrete arch bridge is built (see Figure 7), followed by numerical calculations and structural analysis. The SOFiSTiK structural engineering system is applied in numerical calculations. This software is frequently used in the design and analysis of bridge structures [51,52,53,54,55]. In the present structural analysis, the traffic loads class C are assumed, according to standard PN-S-10030 [56]. This standard was applicable in Poland during the reconstruction design process of the old bridge. The traffic load is composed of q and K types of loads, see Figure 8. The load values of q and K type are equal to 2 kPa and 400 kN (8.P = 8.50 kN = 400 kN), respectively. The distribution of q load (arbitrary distribution) and location of K load have to produce the largest responses in analyzed structural elements. Besides this traffic load, permanent loads are also imposed (dead-weight and bridge equipment’s loads). The shell (SH3 D) and 3 D beam (B3 D) finite elements (FE) are applied in the FEM model of the arch bridge. The finite elements adopted in this study are 4-node isoparametric shell finite elements of DKMQ type and 2-node 3 D beam elements of the Timoszenko type, C0 class with linear shape functions. The mesh independence study of the bridge finite element model is carried out to ensure that the results of an analysis are not affected by changing the size of the mesh. The FEM model of the old concrete arch bridge is meshed by 45,075 shell elements (modeled slab and concrete arch) and 776 3 D beam elements (girders), the model includes 42,223 nodes with and 940 support constraints (Figure 7). It should be noted, that the authors’ experiences in the design and monitoring of bridge structures are also exploited during the construction of the FEM model [57,58]. It can be pointed, that more detailed numerical models are created to investigate the individual structural elements of bridges and comparisons them with laboratory tests [59,60].

### 4.2. Results of Structural Analysis

**Figure A1:**
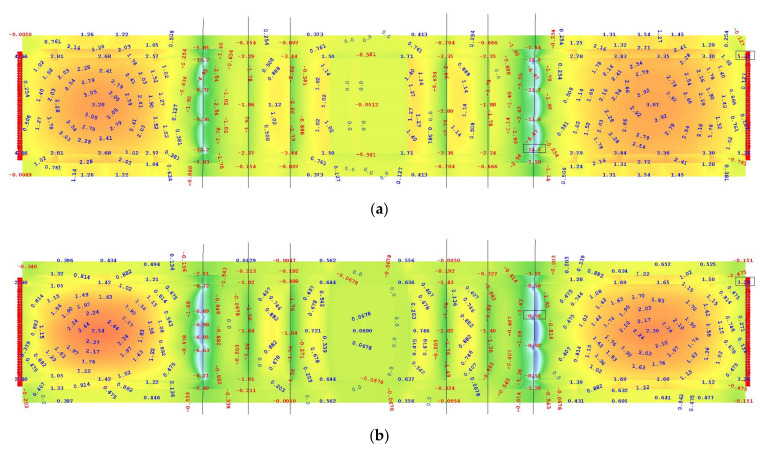
Maps of characteristic values of bending moments *M*xx (kNm) in the deck slab: (**a**) Dead weight; (**b**) bridge equipment loads.

**Figure A2:**
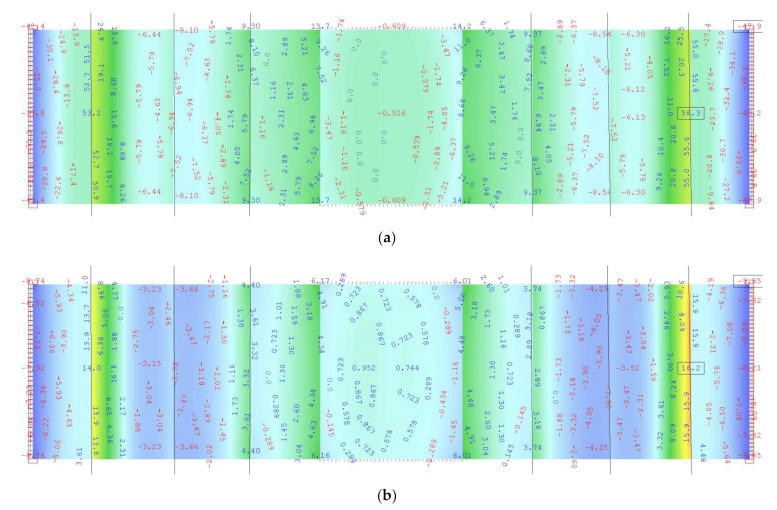
Maps of characteristic values of bending moments *M*xx (kNm) in the concrete arch: (**a**) Dead weight; (**b**) bridge equipment loads.

**Figure A3:**
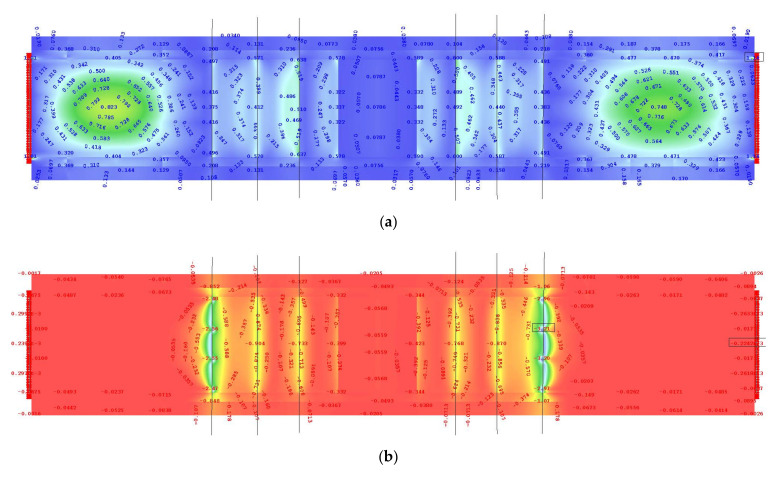

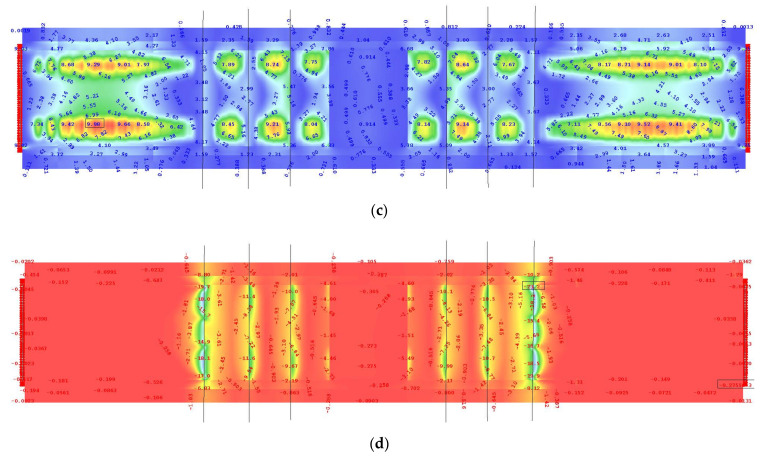
The envelope maps of characteristic values of bending moments *M*xx (kNm) in the deck plate: (**a**) Max Mxx (kNm) under the moving load q of class C; (**b**) min Mxx (kNm) under the moving load q of class C; (**c**) max Mxx (kNm) under the moving load K of class C; (**d**) min Mxx (kNm) under the moving load K of class C.

**Figure A4:**
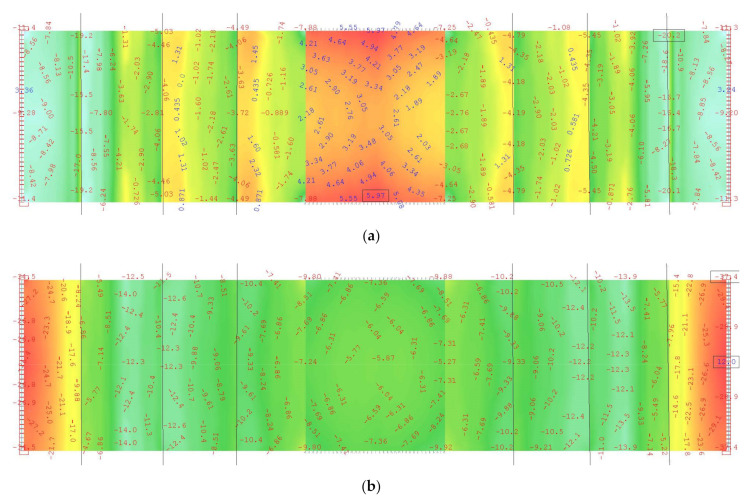

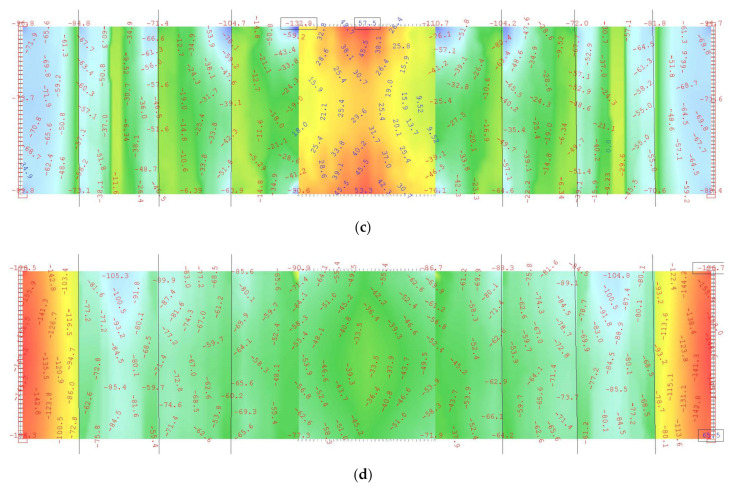
Envelope maps of characteristic values of forces *N*xx (kN) in the concrete arch: (**a**) Max Nxx (kN) under the moving load q of class C; (**b**) min Nxx (kN) under the moving load q of class C; (**c**) max Nxx (kN) under the moving load K of class C; (**d**) min Nxx (kN) under the moving load K of class C.

**Figure A5:**
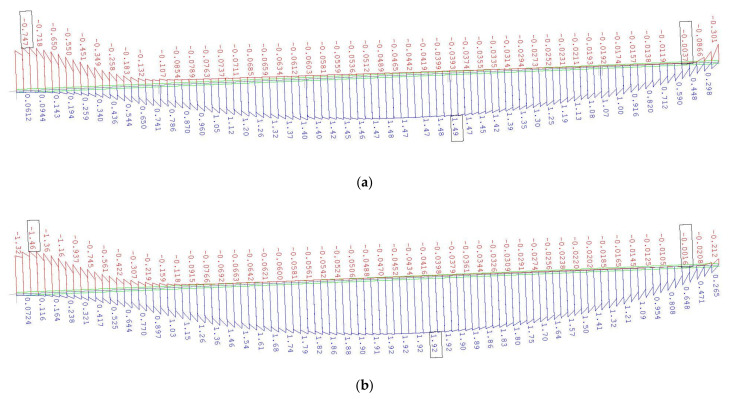
Characteristic values of bending moments *M* (kNm) under the moving load q of class C in the right span: (**a**) Outer girder; (**b**) inner girder.

**Figure A6:**
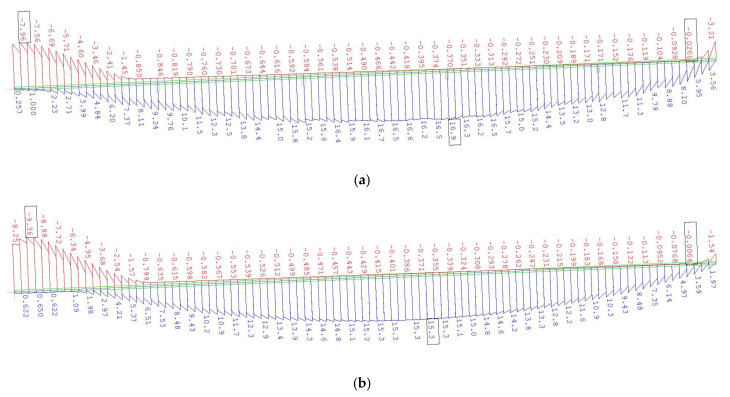
Characteristic values of bending moments *M* (kNm) under the moving load K of class C in the right span: (**a**) Outer girder; (**b**) inner girder.

The numerical analysis allows to evaluate the internal forces acting on the sections and specifically their largest values, which in turns make it possible to define the geometry of the members and later to proceed with the verifications at the Serviceability Limit State (SLS) under the service loads, and at the Ultimate Limit State (ULS) under the ultimate loads. The maps of bending moments in the deck slab and the concrete arch under the dead weight of the structure and loads of the bridge equipment are determined in Figure A1 and Figure A2 respectively. On the other hand, for the moving loads q and K of class C (see Figure 8), the envelope maps of characteristic values of bending moments in the deck slab (see Figure A3) and the envelope maps of characteristic values of forces in the concrete arch (see Figure A4) are presented. In Figure A5 and Figure A6 the characteristic values of bending moments for inner and outer girders are shown. The largest bending moments and axial forces at the chosen, critical cross-sections of a superstructure correspond to the most unfavorable combinations of permanent and live loads according to PN-S-10030 [56], these values are presented in Table 4. The lack of symmetry in the results of internal forces between the right and left span girders results from different spans (see Figure 1). The results for the outer girder and the inner girder are similar in the longer span (right span, see Table 4) where they have a similar height, while in the shorter span (left span) the results differ significantly between the outer girder and inner girder because the girders have different constructional heights.

The site inspection and inventory of old arch bridge make it possible to assess the amount of reinforcement in structural elements, see Table 5. Concrete detector for rebar localization, depth measurement, and size estimation are necessary for the proper estimation of steel reinforcements in the existing old concrete bridge. New technologies, methods, and systems are still developed for the detection of steel rebars in concrete structures [61,62,63,64].

The design yield strength of reinforcement (*f*_yd_) and design compressive strength of old concrete (*f*_cd_) are determined according to specified characteristic yield strength of reinforcement (*f*_yk_), characteristic compressive strength of concrete (*f*_ck_), partial safety factors for reinforcement (*γ*_s_) and concrete (*γ*_c_) material properties. The partial safety factors *γ*_s_ and *γ*_c_ are assumed according to Table NA.2 guidelines in National Annex to PN-EN 1992-1-1 [65]. The characteristic yield strength of steel rebars and the characteristic compressive strength of concrete are specified in laboratory tests.

The largest stresses in reinforced steel and old concrete in representative structural elements of the arch bridge are determined and collected in Table 5. The highest stress in steel rebar’s equals 145.6 MPa, approximately equal to 57% of the design yield strength of reinforcement, *f*_yd_ = 257 MPa, see Table 5. The specified maximal compressive stress in old concrete arch bridge is 9.3 MPa, approximately equal to 96% of the design compressive strength of concrete *f*_cd_ = 9.7 MPa.

**Figure A7:**
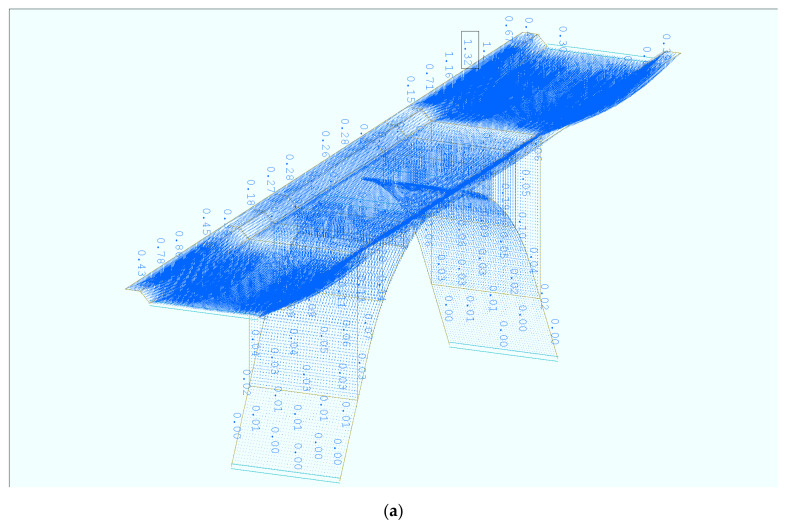

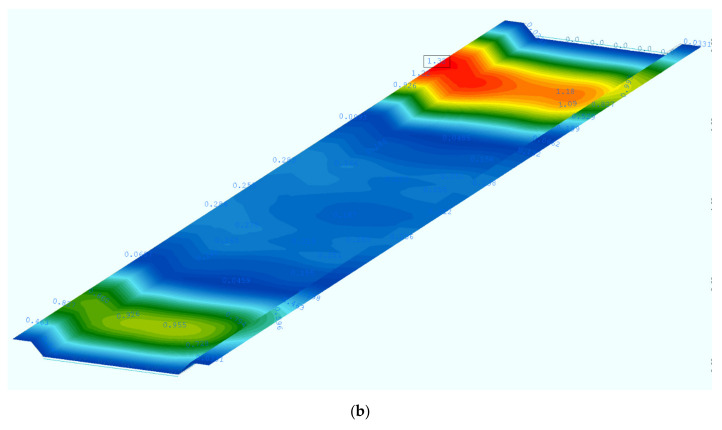
Extreme displacement *f* (mm) under the SLS combination of traffic loadings class C according to PN-S-10030 [56] standard: (**a**) 3 D structure view; (**b**) displacement map on the deck slab.

The largest displacement under the SLS combination of the traffic loadings (see Figure A7) is equal to about 1.3 mm. According to the assumptions of PN-S-10042 [66] standard, the permissible deflection of reinforced concrete beam elements in continuous systems (in this case the largest spans of the analysed bridge, see Figure 1a and Figure A7) is *f*_perm_ = *L*/1000 = (6380 + 2·250)/1000 = 6.88 mm. Determined largest deflections fulfils the SLS condition *f*_max_ = 1.32 mm < *f*_perm_ = 6.88 mm.

Structural analysis indicated that the 95-year old concrete arch bridge structure satisfies SLS and ULS requirements under the traffic loads class C according to PN-S-10030 [56] standard. Sometimes the car traffic on old bridges is not permitted and after reconstructions are able to meet the requirements for footbridges only [67]. The structural analysis confirmed the validity of the adopted design solutions. On top of the existing old deck structure, a new deck slab with pavement covers is designed and the entire surface of the old concrete is protected with repair mortars. The task of the new deck slab is not only to strengthen the structure but also to increase the resistance to present environmental influences and also the shear capacity of the deck slab. The higher strength class of concrete is designed and used to fulfill the EN 206 [47] standard requirement of the present exposure classes.

All newly erected bridges and old rebuilt bridges need a detailed assessment and dynamic analysis. In this paper, this analysis is not included but the dynamic analysis is performed to properly assess the properties of the 95-year-old arch concrete bridge. It should be noted, that improper design of bridge structure cause the risk of excessive structural vibrations throughout the operation [68,69,70,71,72,73].

## 5. Conclusions

A 95 year-old concrete arch bridge in Jagodnik (northern Poland) is examined in this paper, from the properties of the materials—by testing samples in a lab—to the structural behavior—by finite-element modeling, with the following results:

- The investigated core samples of the 95-year-old concrete exhibit a high compressive strength scatter from 14.9 MPa to 29.7 MPa, see Table 1. These results serve as evidence of the very non-homogenous distribution of concrete strength in the old concrete arch bridge.
- The concrete specimens tested in compression show that there is a large number of impurities in the old concrete (i.e., pieces of timber and clay, coarse aggregates, large stones) with cavities and soft pockets. The scatter in the mechanical properties is, therefore, due to either very low quality-control in the building phase or very poor concrete technology based on portable concrete mixers and hand-made proportioning of concrete constituents.
- The mean dry density of the original concrete equals 2165 ± 8 kg/m^3^, see Table 1. The old concrete satisfies the conditions for normal-weight concrete category according to the EN 206 standard [47] and the ACI 318-19 code [48].
- The in-situ compressive cube strength *f*_ck,is,cube_ equals 16.9 MPa, according to EN 13791 [41] standard. Calculated and applied in structural analysis, the design compressive strength of 95-year-old concrete *f*_cd_ equals 9.7 MPa.
- The reinforced steel tensile tests confirm that the characteristic yield strength specified in laboratory tests (*f*_yk_ = 291 ± 7 MPa) corresponds to the guidelines [36] issued in the time of the 95-year-old arch bridge construction.
- The highest stress in steel rebars of structural cross-section is approximately equal to 57% of the design yield strength of reinforcement, the maximum compressive stress in the original parts of the arch bridge is approximately equal to 96% of the design compressive strength of concrete.
- The largest displacement under the SLS combination of the traffic loadings satisfies the SLS condition according to o PN-S-10042 [66] standard. The determined largest deflections are five times less than the permissible deflection.
- The structural analysis confirmed the validity of the adopted design solutions. On top of the existing old deck structure, a new deck slab with pavement covers is designed and the entire surface of the old concrete is protected with repair mortars. The task of the new deck slab is not only to strengthen the structure but also to increase the resistance to present environmental influences and also the shear capacity of the deck slab. The higher strength class of concrete is designed and used to fulfill the EN 206 [47] standard requirement of the present exposure classes.
- The 95-year-old arch bridge satisfies bearing capacity (ULS) and SLS conditions, capable of carrying designed traffic loads class C according to PN-S-10030 [56] in extended working life.

This paper provides scientists, engineers, and designers with experimental and structural assessments of the 95-year-old concrete. The study confirms the old arch bridge ability to carry newly designed traffic loads and helped give him a new structural life and extend his working life. Lots of existed old and historic bridge structures require restoration and reconstruction, which require not only a sound financial plan and the expertise of civil engineers, but—more often than not—the expertise of the scientific community, for an appropriate assessment of structural safety. Old bridge structures should return like the Phoenix from their ashes and recovery their past appearance. The protection of old bridges coincides with the preservation of their cultural heritage.

## Figures and Tables

**Table 1:** Concrete compressive strength and dry density.

Specimens No.	Compressive Strength MPa	Dry Density kg/m^3^
Results Obtained by Ambroziak et al. [30]	14.9	2166
	20.0	2171
	19.1	2132
	18.6	2184
	22.0	2155
	19.9	2064
	19.5	2180
	16.1	2174
	18.9	2147
1_1	24.3	2157
1_2	21.6	2186
1_3	24.5	2207
2_1	17.1	2173
2_2	25.3	2170
3_1	29.7	2193
3_2	17.0	2179
mean	20.5 ± 1	2165 ± 8
median	19.7	2172.1

**Table 2:** Concrete strength depending on the amount of cement in 1 m^3^ aggregate (according to guidelines given in [36]).

The Amount of Cement (kg) in 1 m^3^ of Aggregate	Forecast Compressive Strength MPa (kg/cm^2^)
500	19.6 (200)
400	16.7 (170)
300	13.7 (140)
200	9.8 (100)
100	5.9 (60)

**Table 3:** Concrete strength in MPa (kg/cm^2^) depending on the amount of cement in 1 m^3^ of ready concrete on the degree of liquidity and the volume ratio of aggregate (according to guidelines given in [37]).

Volume Ratios	The Amount of Cement (kg) in 1 m^3^ of Concrete Mix	Liquid Consistency	Plastic Consistency	Rammed Consistency
Sand to gravel 1:1 or sand to stone gravel 1:0.8	200	0 (0)	2.9 (30)	5.9 (60)
	300	4.9 (50)	8.8 (90)	11.8 (120)
	400	9.8 (100)	13.7 (140)	15.7 (160)
Sand to gravel 1:2 or sand to stone gravel 1:1.6	200	3.9 (40)	8.8 (90)	11.8 (120)
	300	9.8 (100)	13.7 (140)	15.7 (160)
	400	13.7 (140)	17.7 (180)	19.6 (200)

**Table 4:** Characteristic and design value of internal forces under designed loads.

Loads	Right Span		Left Span		Slab and Arch		
	Outer Girder	Inner Girder	Outer Girder	Inner Girder	Center Deck Plate	Arch	
	*M* kNm	*M* kNm	*M* kNm	*M* kNm	*M* kNm	*M* kNm	*N* kN
L1—characteristic load by dead-weight	9.8	10.0	8.4	3.7	1.4	56.3	560.4
L2—characteristic load by bridge equipment’s	5.1	6.1	4.5	2.5	0.9	16.2	115.0
L3—characteristic live load, standard load q type	1.5	1.9	1.4	0.8	0.5	6.8	37.4
L4—characteristic live load, standard load K typewithout the dynamic factor ϕ	17.0	15.3	16.0	6.5	9.2	38.7	196.8
Sum: L1 ÷ L4	33.4	33.3	30.3	13.5	12	118	909.6
dynamic factor ϕ	1.318		1.324		1.325	1.284	
design value with dynamic factor ϕ	55.1	53.8	50.4	22.3	21.7	172.6	1280.3

**Table 5:** Materials characteristic and calculated extreme stresses in concrete and steel rebar’s.

Properties	Left Span		Right Span		Slab and Arch	
	Outer Girder	Inner Girder	Outer Girder	Inner Girder	Deck Plate	Arch
Steel rebar’s (bottom)	6#20		6#20		14#12/m	5#12/m
Design yield strength of reinforcement	*f*_yk_ $=291\;\mathrm{MPa},\;\mathrm{thus}\;f_{\mathrm{yd}}=\frac{f_{\mathrm{yk}}}{\gamma_{s}}=\frac{291}{1.15}=257\;\mathrm{MPa}$					
Design compressive strength of concrete	*f*_ck,is,cube_ $=16.9\;\mathrm{MPa},\;\mathrm{thus}\;f_{\mathrm{cd}}=\alpha_{\mathrm{cc}}\frac{f_{\mathrm{ck}}}{\gamma_{c}}=1.0\frac{0.8\cdot 16.9}{1.4}=9.7\;\mathrm{MPa}$					
Determined largest stress in steel rebar’s, MPa	32.7	31.9	38.1	16.8	78.6	145.6
Determined highest stress in concrete, MPa	2.2	2.2	3.0	1.3	3.3	9.3

## Data Availability

All laboratory test results (data) are included in Table 1 in the present paper.

## Appendix A. FEM Calculation Results

The chosen results of internal forces used in the verification and design process (see Table 4) are presented in Figure A1, Figure A2, Figure A3, Figure A4, Figure A5 and Figure A6. In Figure A7 extreme displacement under the SLS combination of traffic loadings is given.
